# Books for Hospitals

**Published:** 1887-09-10

**Authors:** 


### BOOKS FOR HOSPITALS.

By One of the Crowd.

"Oh, yes; we get a great many books, and magazines, and newspapers sent us, and I am bound to say we get among them an immense amount of rubbish."

The speaker is the Rev. W. H. W. Casely, the genial chaplain of St. Thomas's Hospital, and I have reason to believe that this statement of his might be made for all the larger hospitals of the kingdom. As he spoke he led the way to his private apartment in order that I might see for myself the heterogeneous collection that had recently been received at St. Thomas's, but which had not found its way into the wards. One side of his room was stacked with hundreds of publications, good, bad, and indifferent, and which, as Mr. Casely pointed out, very fairly illustrated this particular phase of public charity.

To begin with, at the inner end of the room stands a sack, into which are promptly stuffed all sorts of sensational trash in the shape of pictorial sheets, stories of the Jack Sheppard type, vulgar scurrilities, coarse and objectionable prints of all kinds, and newspapers dismally out of date.

It is of no use to send this sort of rubbish to the hospitals. These matters are generally looked after by the chaplains, who are usually men of anything but bigoted character or narrow sympathies. They have to do with people of all sorts of creeds, and with tastes as diversified as their ailments. But any person of sense or sensibility can see that many of the catchpenny publications of the day would be wholly out of place on beds of sickness and suffering, and of course they are no sooner received than they are tucked into the wastepaper-bag.

Then again, there are received many other things to which no such objections can be raised, but which one would think can only be sent as grim jokes—bundles of old music, for instance, works on algebraic formulæ, and packages of life-insurance tables. No doubt actuaries and mathematicians do occasionally put into dock, as it were, in these institutions, and now and again, perhaps, a musician who can find pleasure in a musical score apart from its performance; but yet these are hardly the kind of thing for a hospital library. Much of Mr. Casely's stock here is pretty evidently the turnings-out of cupboards and odd corners, the gift of good people who have pleased themselves with the idea that they are doing a charitable act, whereas they are merely relieving their premises of lumber.

Again, it is not reasonable to suppose that the chaplain or any other responsible officer of a great hospital, the very air of which is heavy with sorrow and suffering, accidents and death, will circulate among patients the publications of those who are notoriously hostile to religion. Your atheistic materialism may be all very fine for you, my friend, in the heyday of your strength and the pride of your able-bodied manhood. But these poor mortals! What is it for them? Writhing in anguish, smitten with hopeless disease, maimed and mangled, face to face with death in some of its most appalling forms—however smart and clever your book may be, how *could* any man of heart or kindly feeling put it into the hands of a patient in a place like this, if the tendency of the book is to break down any little faith he may have in the pitiful Fatherhood of God, and the hope of a happier life hereafter? If your view of things is the right one, one would not obtrude it here. Common-sense and sound philosophy would say: "It is a dreadful truth; here, at least, let us try and forget it. Hush it up and smuggle it out of sight." It is strange that there should be people who deem it worth while to send anti-christian publications for use in hospitals; but there are such people.

Then there are those who send very good books in a shockingly bad condition—dog-eared, dirty, torn, coverless, raggamuffin-looking volumes, that the donors themselves would be heartily ashamed of if it were possible that they could see them on the lily-white beds of any well-conducted hospital. Ragged and dirty books are not used, and, as a rule, people should not send works that are incomplete—books with some of their pages missing, or a single volume of a tale published in two or three volumes. In short, they should send to a hospital only that which has intrinsic value, and is capable of affording wholesome amusement, or useful information, or moral benefit. At the same time it should be remembered that the patients in these places often include persons of superior education; and by no means let it be understood that only that which is light and trifling and comparatively superficial is desirable.

There should be works of high-class solid interest—standard works of all kinds; good fiction is especially desirable; all the respectable magazines are welcome, and if sent in complete sets, the authorities are usually glad to go to the expense of binding them,—though it need hardly be said that they prefer to receive them well bound; fresh newspapers are very acceptable, especially the respectable illustrated ones. Everybody likes to see them, and there are a good many patients who can be interested in pictures, when they are too feeble or too ignorant to be able to read letterpress. Last, but by no means least in importance, are books and periodicals and picture scrap-books for the children. Most hospitals have some children in them, and in making up a parcel of books for one of these institutions, the children should not be forgotten. Send them something bright and pretty and good—but not "goody-goody," please.

There is one other suggestion that it may be well to make. Where there are books there ought to be book-cases. Most of the wards in our hospitals are lacking in these conveniences, and anyone who would like to do an act of permanent and benevolent usefulness might find many a less judicious scope than in presenting a neat book-case to a hospital ward. A very gratifying object in one of the wards of St. Thomas's Hospital is a piece of furniture of this description—a handsome little mahogany glass-fronted book-case, which, says a brass plate on the front, was presented, together with 150 volumes, by a patient in grateful memory of benefits received in 1879. Not every patient could thus manifest his gratitude, but a good many might, one would think, present a nice volume.

It may be well just to add that the Honorary Secretary of the literature branch of the Kyrle Society has recently issued an appeal for books, periodicals, and papers for distribution to public institutions that may be in need of them. Among such institutions hospitals and infirmaries hold, of course, a prominent place, and anyone having anything of the kind to give, but being in doubt as to the institution most in need, cannot do better than despatch the parcel to this excellent organisation at 14, Nottingham Place, W. They may be quite sure then that their books and papers will not go where they are not particularly needed.

